# Rapid and reversible fish skin mucosal microbiota shift toward environmental microbial communities within a day

**DOI:** 10.1128/spectrum.00522-26

**Published:** 2026-07-16

**Authors:** Chie Imamura, Yoshikazu Furuta, Hidenori Tanaka

**Affiliations:** 1 Toyota Central R&D Labs., Inc., Aichi, Japan; Northwest A&F University College of Animal Science and Technology, Yangling, Shaanxi, China

**Keywords:** fish skin microbiome, environmental translocation, microbial plasticity, host-environment interaction, freshwater microbiome, 16S rRNA amplicons

## Abstract

Fish are constantly exposed to the surrounding environmental water, and fish skin mucosal microbiota is considered to be strongly influenced by the microbiota of the surrounding environmental water. However, the temporal dynamics of these changes, how rapidly and to what extent the fish skin microbiota becomes similar to the environmental water microbiota, remain unclear. Here, we repeatedly transferred *Tanakia lanceolata* between an artificial aquaponic system and a pond resembling a natural environment to characterize temporal changes and reproducibility. 16S rRNA gene amplicon analysis revealed that the fish skin microbiota underwent rapid community restructuring within 1 day after transfer and became more similar to the microbial community composition of the destination water than to that of the pre-transfer water. This pattern was consistently observed across two independent experiments involving multiple transfers. The main taxa in the skin belonged to the phylum *Proteobacteria. Gammaproteobacteria* were abundant in the aquaponics system, whereas *Alphaproteobacteria* and *Actinobacteria* were also abundant in Fukada Pond. The core skin microbiota for each environment differed markedly between the two environments and exhibited increases, decreases, or replacement following transfer. Furthermore, alpha diversity of both skin and environmental water microbiota increased in natural ponds and decreased in the aquaponic systems. These findings provide new insights into host-environment microbial interactions and demonstrate the ecological plasticity of fish-associated microbiota in response to rapid environmental changes.

**IMPORTANCE:**

With respect to the response of the fish skin microbiota to changes in environmental water, high-resolution short-term temporal data would allow for the assessment and management of fish health during short periods, such as transfers between tanks. Here, we revealed that fish skin microbiota is highly plastic and rapidly and reversibly restructures itself in response to environmental changes. Two experiments reproducibly demonstrated that repeatedly transferring fish between different environments leads to the reconstruction of the skin microbiota within 1 day post-transfer, resulting in a composition more similar to that of the destination’s environmental water microbiota than to that of the pre-transfer water. These results indicate that fish skin microbiota respond dynamically to the surrounding microbial environment. These findings provide a foundation for understanding host-environment microbial interactions in fish health management and aquaculture system design.

## INTRODUCTION

Fish are exposed throughout their lives to various environmental stresses, such as water temperature, pH, dissolved oxygen, harmful substances, and pathogens (1). In particular, during the movement or transport of cultured or ornamental fish, abrupt changes in the rearing environment impose major stress, which is known to weaken or cause mortality in fish. Therefore, understanding the effects of stress is important for maintaining and managing fish’s health during aquaculture and transportation. Environmental stressors in aquatic systems include both physicochemical and biological stresses, and many studies have examined the effects of physicochemical stressors on fish (2–5). There are also reports that differences in physicochemical parameters of water are significantly correlated with differences in the community structure (β diversity) of fish skin microbiota (6) and that disinfectants used in aquaculture markedly alter fish skin microbiota, indicating that environmental changes directly affect the composition of the skin microbiota (7).

The invasion of pathogens and the effects of non-pathogenic bacteria can be cited as biological stressors. The skin mucus of fish is the first defensive barrier against pathogens and contains several immune components (8). When the balance of the skin microbiome is disrupted, such as during pathogenic infection, there is a risk of increased opportunistic pathogens and immune response induction (9–13).

In addition, there is concern that not only pathogens but also rapid changes in the surrounding environmental water microbiota (non-pathogenic bacteria), such as those accompanying environmental transfers, may disrupt the balance of the fish skin microbiota and adversely affect fish health, including a decline in immune function. Analysis of data from 36 studies on the skin microbiota and rearing environments of 98 fish species worldwide, comprising a total of 1,922 samples, revealed that differences in the rearing environment exert a small but significant effect on the composition of the skin microbiota (approximately 12% of the total variance in community structure) (6). In aquaculture ponds, although dominant taxa differ between fish skin and rearing water, they share common taxonomic groups (14). Moreover, biofilms formed in artificial hatchery tanks strongly influence the mucosal microbiota of fish reared in these systems (15, 16).

In aquaculture and related settings, when the rearing environment of a fish is transferred to another environment, the microbiota of the environmental water pre- and post-transfer differs; therefore, the fish skin microbiota may change significantly as described above. Although detailed investigations are expected to be conducted on the relationship between the microbiota of fish skin and that of the environmental water when fish are transferred to different water environments, studies on fish skin microbiota following changes in the rearing environment are limited. In a transfer experiment in which wild Atlantic salmon fry and farmed fry were exchanged and reared for 6 months, it was reported that the skin microbiota of the fish did not resemble the microbiota of the destination environment and that the water microbiota differed from that of the gut and skin (17). Experiments transferring roach between brackish and freshwater show that their skin microbiota rapidly shifts within 1 week in freshwater, and then their skin microbiota tends to approach the skin microbiota of fish residing in brackish water (18, 19). In both cases, no information was available on how quickly the microbiota changed after transfer. If much shorter-term time-series information were available, it would be possible to manage health during short transfer periods between tanks and rapidly assess the health status of fish. In addition, whether the surrounding environmental water microbiota and the fish skin microbiota resemble each other remains controversial and requires further elucidation.

To investigate how the fish skin microbiome changes over time and to what extent it becomes similar to that of the surrounding environmental water, in this study, we examined the temporal dynamics of the microbiota of fish skin and environmental water when *Tanakia lanceolata* was repeatedly transferred between two different environments using a metabarcoding approach targeting the 16S rRNA gene. To the best of our knowledge, this is the first detailed time-series study examining whether the fish skin microbiota becomes similar to the environmental water microbiota when fish are transferred to different water environments, and whether such similarity also occurs when the transfers are repeated.

## MATERIALS AND METHODS

### Overview of environmental translocation experiments

The experiment was conducted between the aquaponic system installed at the entrance of Toyota Central R&D Labs, Inc. in Nagakute City, Aichi Prefecture, and Fukada Pond, located within the adjacent Toyota Physical and Chemical Research Institute premises (Fig. S1). Aquaponics integrates soilless plant hydroponics with fish culture (20, 21), wherein fish waste is microbially converted into nutrients for plant growth, while water is simultaneously purified and recirculated. This circular and sustainable design aligns with the ongoing SDG-oriented initiatives (22). The experimental aquaponics system consisted of a ~300 L water tank connected to five inclined wall-greening panels (~2.5 m high) arranged to mimic a vegetated slope. Water was distributed to each panel and returned to the tank via a recirculating loop three times per day (30 min each), 5 days per week. In the water tank, continuous water flow was maintained using a submersible pump without aeration. Aquatic plants were grown within the panels, and the fish community included several native Japanese cyprinids, including slender bitterlings (*Tanakia lanceolata*), *Gnathopogon*, and other related species.

Fukada Pond, located adjacent to the facility, is a rainwater retention pond that was dredged in 2017 to improve water quality. The pond currently supports diverse fish communities, including carp (*Cyprinus carpio*), crucian carp (*Carassius* spp.), gobies, *Gnathopogon*, *Tanakia*, and other native species.

To minimize stress to the fish, DNA was collected from the fish using a non-invasive sampling method in which the fish’s skin was gently swabbed two or three times. The experiment was approved by Toyota Central R&D Labs, Inc., with approval numbers 22A-10 and 22B-04. All procedures were performed in accordance with ASAB/ABS animal welfare guidelines.

### Experimental design and fish transfer schedule

**Fig 1:**
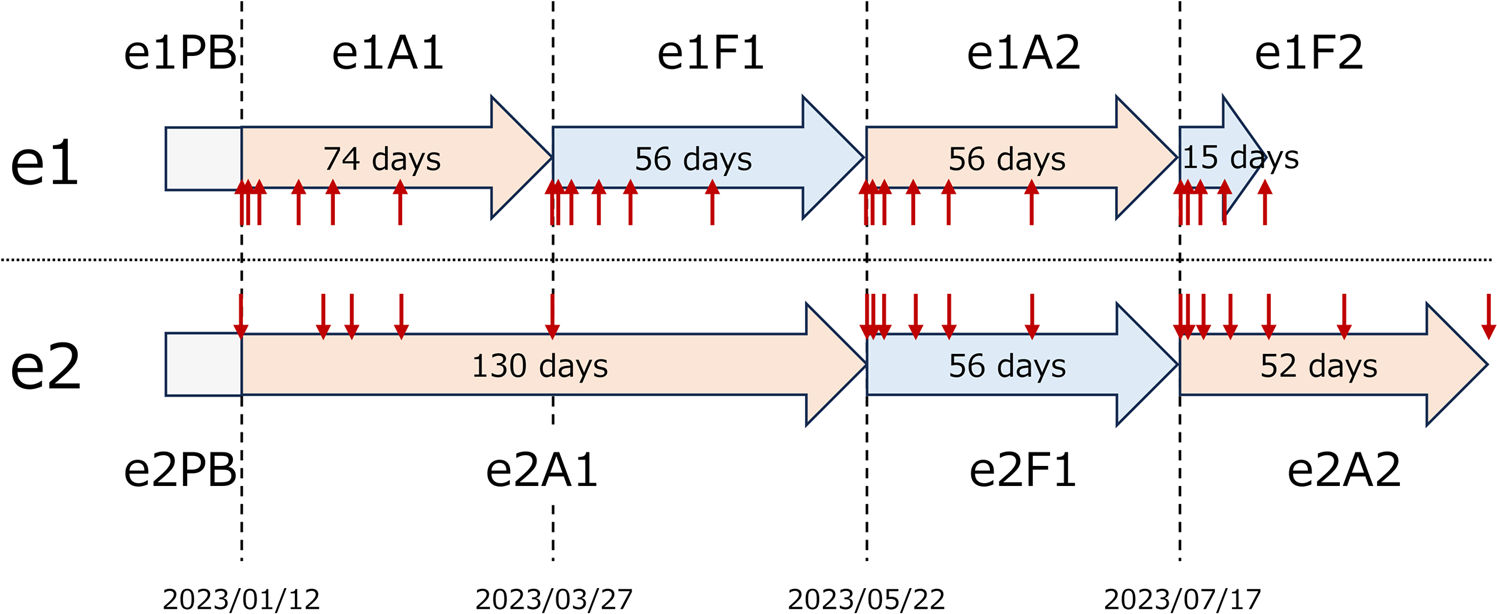
Overview of the fish translocation experiments between the aquaponics system and Fukada Pond. Two independent experiments (e1 and e2) were conducted with repeated moves. Sampling days are indicated by red arrows; sampling details at each translocation interval are in Table S1. A total of 117 skin mucus samples and 39 environmental water samples were used, based on sampling over 23 days in e1 and 18 days in e2 (Table S1). The sample IDs of the total 156 samples were named by combining “Experiment_day_group_fish with the number of the biological replicate.”

Two independent experiments were conducted (Experiment 1, e1; Experiment 2, e2) (Fig. 1). Six *Tanakia lanceolata* individuals were purchased from a.a.c. Co., Ltd. (Osaka, Japan) and divided equally between the two experiments (three fish per experiment). Three fish were placed in a cylindrical keep net (“sukari”; 30 cm diameter, 30 cm depth), and the same three individuals were sampled longitudinally at all the time points. The experiment was conducted between January and September 2023.

#### Experiment 1 (e1)

The fish were first moved from the plastic bag at purchase (e1PB) to the aquaponics system, where a translocation interval of 74 days (e1A1) was set. Thereafter, the fish were transferred to Fukada Pond, and a translocation interval of 56 days (e1F1) was set. The same transfers were then repeated, establishing translocation intervals of 56 days in the aquaponic system (e1A2) and 15 days in Fukada Pond (e1F2). During e1F2, from day 3 to day 15, one of the three fish died, and sampling was therefore conducted using the remaining two fish.

#### Experiment 2 (e2)

The fish were similarly transferred between the two locations, but the number of transfers and intervals differed; the first translocation interval in the aquaponic system was 130 days (e2A1), followed by 56 days in Fukada Pond (e2F1), and finally 52 days in the aquaponic system (e2A2). During e2A1, from day 13 to day 36, due to a sampling error, only two individuals per group were used for the analysis.

Sampling of both the environmental water and fish skin mucus was performed immediately before each transfer and subsequently at approximately 1 day, 3–4 days, 1 week, 2 weeks, 1 month, and 2 months (or on the final day) after transfer, as indicated by the red arrows in Fig. 1. Fish were fed once weekly (mainly Friday evening) with *Saki-Hikari* Slow-Sinking Food for Goldfish Fry (Kyorin, Japan). To minimize dietary effects, at least 3 days elapsed between feeding and sampling.

### Sampling of environmental water and fish skin mucus

Environmental water samples were collected from each site in sterile disposable beakers, transported to the laboratory on ice within 30 min, and filtered (200 mL) through 0.2-μm Analytical Filter Funnels (Nalgene). Filters were then immersed in 1 mL of DNA/RNA Shield (Zymo Research) and stored at –80°C until DNA extraction.

Fish skin mucus was sampled using DNA/RNA Shield Collection Tubes (Zymo Research) by gentle swabbing from the head to the tail (two to three passes). Each swab was immediately suspended in 1 mL of DNA/RNA Shield and frozen at –80°C within 30 min of collection.

### DNA extraction

After thawing, each sample suspension was treated with Proteinase K (1/40 vol; Zymo Research) at 55°C for 15 min to degrade the mucins, followed by DNA extraction using the ZymoBIOMICS DNA Miniprep Kit (Zymo Research), according to the manufacturer’s instructions. DNA concentrations were quantified fluorometrically using the QuantiFluor ONE dsDNA System (Promega).

### 16S rRNA gene sequencing

NGS library preparation and sequencing of the 16S rRNA gene (V3–V4 region) were outsourced to the Bioengineering Laboratory Co. Ltd. (Kanagawa, Japan). Two-step-tailed PCR amplification was performed using primers 1st-341f_MIX (ACACTCTTTCCCTACACGACGCTCTTCCGATCTNNNNNCCTACGGGNGGCWGCAG) and 1st-805r_MIX (GTGACTGGAGTTCAGACGTGTGCTCTTCCGATCTNNNNNGACTACHVGGGTATCTAATCC). Sequencing was conducted on an Illumina MiSeq platform using the MiSeq Reagent Kit v3 (Illumina) with 300 bp paired-end reads.

Raw sequence reads were processed using the Microbial Genomics Module of the CLC Genomics Workbench (Qiagen). Adapter and primer sequences (forward: CCTACGGGNGGCWGCAG; reverse: GACTACHVGGGTATCTAATCC) were trimmed, low-quality reads (quality limit 0.05) and fragments shorter than 15 bp were removed, and paired-end reads were merged with a minimum overlap of 20 bp. Operational taxonomic units (OTUs) were clustered at 99% sequence similarity with the SILVA SSU Ref database (version 138.1; 99% identity; https://www.arb-silva.de/documentation/release-1381/). The OTUs annotated as chloroplasts were removed. In addition, OTUs with an abundance of 0.005% or less of the total reads were excluded from the analysis. OTU abundance tables are shown in Table S2a (e1) and Table S2b (e2).

### Evaluation of microbial community similarity between environmental water and fish skin mucus

To quantify the similarity between microbial communities from the environmental water and fish skin surfaces, pairwise dissimilarity was calculated using the theta dissimilarity index (TDI [23, 24]) derived from the thetaYC index. The TDI value (= 1 − thetaYC) ranges from 0 to 1, where 0 indicates identical community structures, and 1 indicates completely distinct communities. TDI is widely used in microbial community analyses, particularly in gut microbiota studies, to compare differences in community composition.

TDI values were calculated in R Statistical Software (v4.2.1 [25]) based on the relative abundance of each OTU from paired fish skin and environmental water samples collected on the same sampling date according to the following relationship:


$$TDI=1-thetaYC=1-\frac{\sum_{i}a_{i}b_{i}}{\sum_{i}(a_{i}-b_{i}{)}^{2}+\sum_{i}a_{i}b_{i}}$$

where *a_i_* and *b_i_* represent the relative abundance of OTU *i* in communities A and B, respectively.

To compare the TDI values between the experimental conditions, four predefined pairwise contrasts (fish vs water in each translocation interval) were tested using the Wilcoxon rank-sum test (two-sided). To control for the false discovery rate across these four pairwise tests, the resulting *P* values were adjusted using the Benjamini–Hochberg method.

### Statistical analysis of diversity indices and visualization methods

Alpha diversity indices (Shannon entropy [26]) were calculated using the CLC Genomics Workbench (QIAGEN). Differences in alpha diversity between environments (aquaponics vs Fukada Pond) were evaluated using the Kruskal–Wallis *H* test. Beta diversity relationships were visualized using principal coordinate analysis (PCoA) based on Bray–Curtis dissimilarities (27) in CLC.

Stacked bar plots of the microbial community composition were generated in CLC. Boxplots of the TDI values were created using the ggplot2 R package (v4.0.1 [28]). Scatter plots were produced in Microsoft Excel, and Venn diagrams were constructed using the Venn R package (v1.12 [29]). Statistical significance was defined as *P* < 0.05, unless otherwise specified.

## RESULTS

### Day-after-transfer comparisons of fish skin microbiota

Read counts for each sample in e1 and e2 are shown in Table S3. The number of reads obtained was 29,578–234,864. They were not subsampled to a common depth. After excluding rare OTUs with an abundance of 0.005% or less of the total reads, the number of OTUs decreased from 17,121 to 1,633 in e1 and from 15,650 to 1,839 in e2. These remaining OTUs were used for the following analyses.

**Fig 2:**
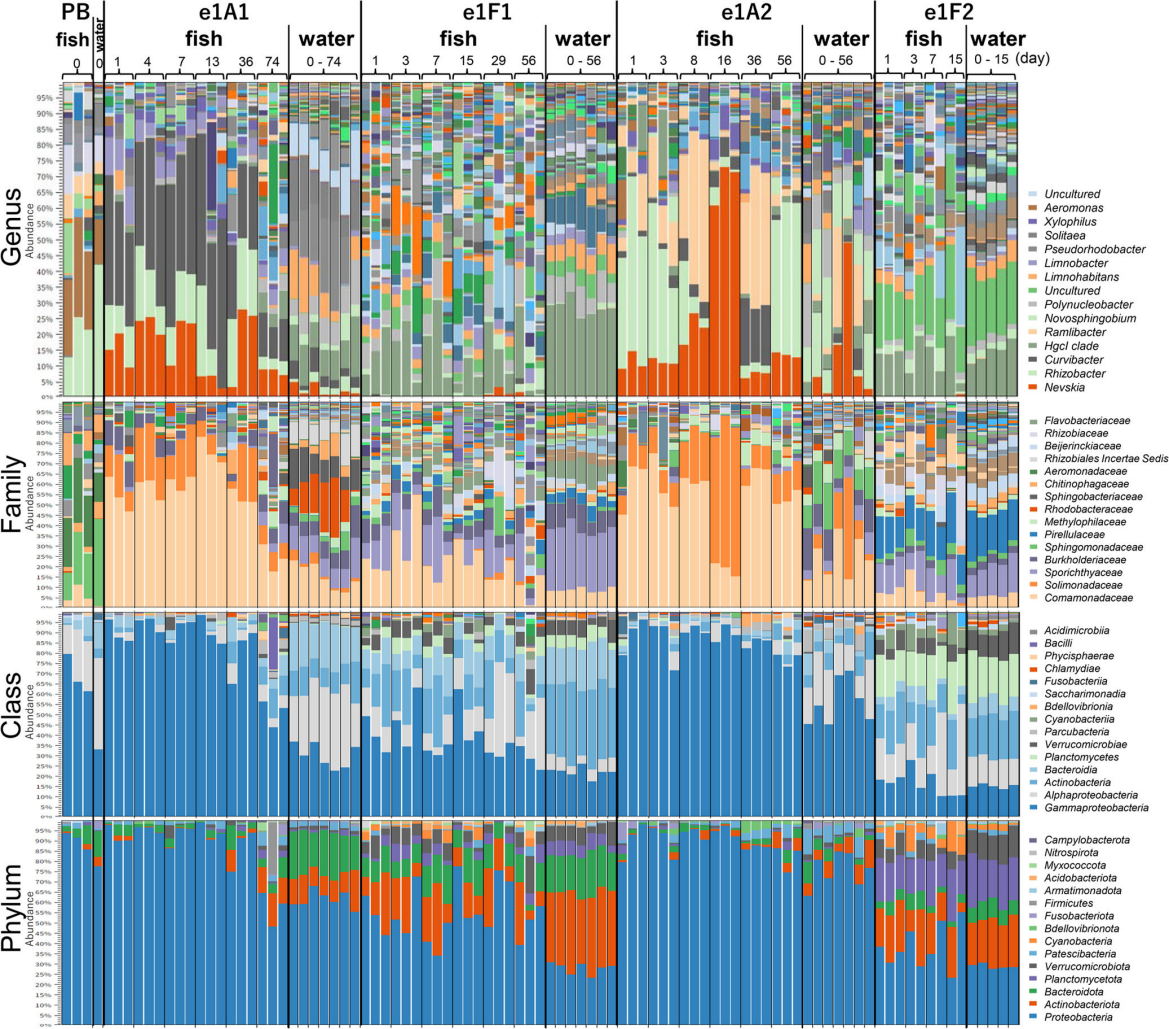
Community composition (genus/family/class/phylum) of water and fish skin across all stages of e1 (e1PB → e1A1 → e1F1 → e1A2 → e1F2).

**Fig 3:**
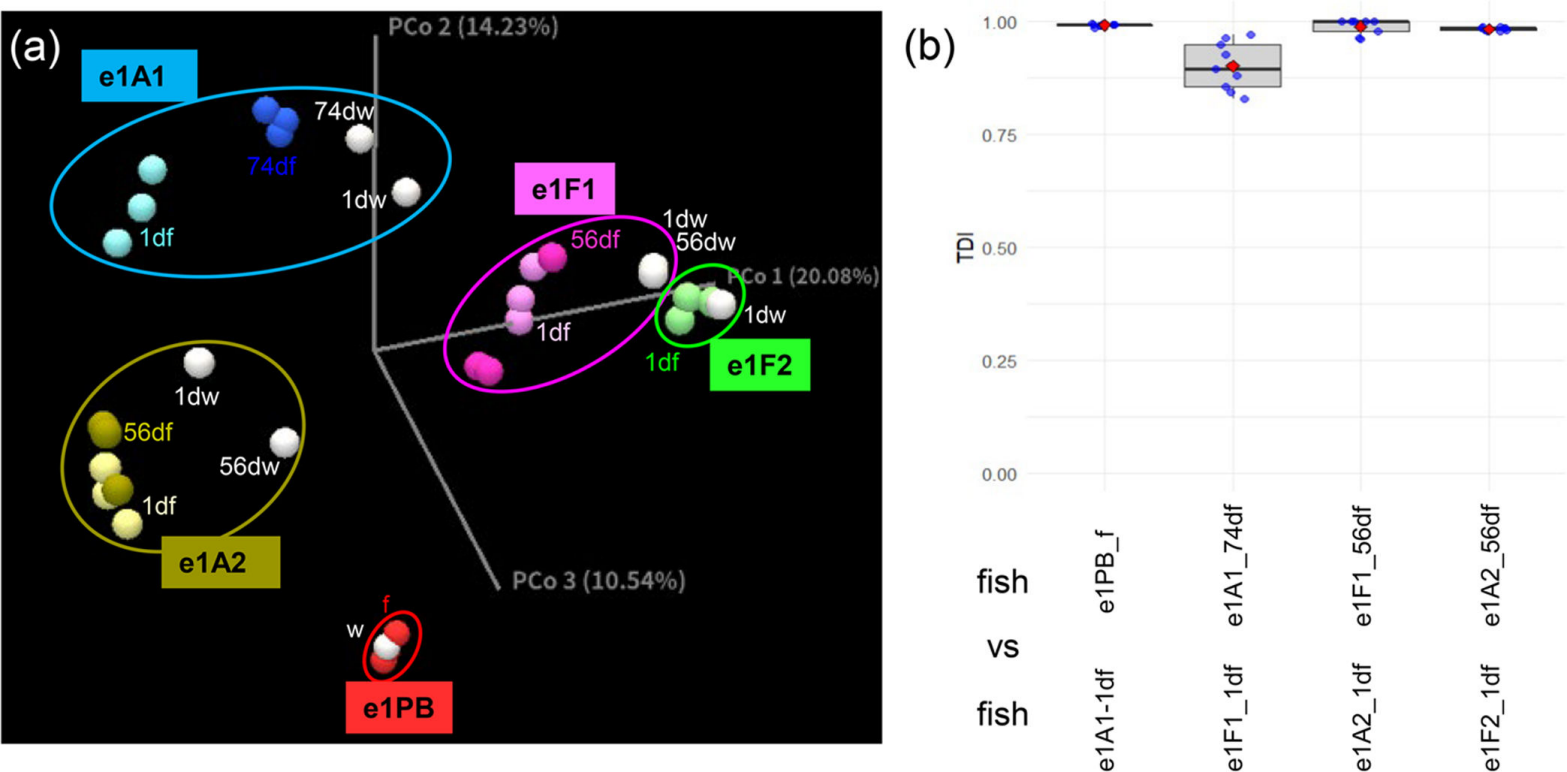
(**a**) PCoA showing shifts in fish skin microbiota after each translocation. The trajectories indicate that, after transfer, the fish skin microbiota shifts away from the pre-transfer state and toward the microbial community of the destination water. Importantly, the post-transfer skin microbiota is positioned closer to the destination water microbiota than to the pre-transfer water microbiota, indicating a directional change in community structure. (**b**) Corresponding theta dissimilarity index (TDI; 1 − thetaYC) for comparisons of fish skin microbiota before and after transfer. Calculated TDI values are indicated by blue circles. Mean (red diamond) and median (black line) are shown. A1, 1st aquaponics; F1, 1st Fukada Pond; A2, 2nd aquaponics; F2, 2nd Fukada Pond; d, day; f, fish skin; w, water.

The microbiota composition at the genus, family, class, and phylum levels in experiment e1 is shown in Fig. 2. The fish skin microbiota differed among the translocation intervals, with large differences observed between the aquaponic and Fukada Pond samples. In addition, genus-level composition showed substantial variability among samples within the same translocation interval (e.g., e1F1 in Fig. 2), indicating that responses were not uniform across individuals. Notably, the composition of the microbiota began to change on the first day after each transfer. Therefore, we created Bray–Curtis PCoA plots to compare the microbiota of fish skin and environmental water on the day immediately before transfer (the final day in the pre-transfer environment) and the day after transfer (Fig. 3a). At each translocation interval, the microbiota of the skin and environmental water on the first and last days were positioned close to each other. Examining the changes in the fish skin microbiota associated with the transfer, we observed that in the first transfer, the initial position of e1PB shifted away and moved toward the aquaponic environmental water plots of e1A1 (1dw, 74dw). In the subsequent transfer from e1A1 to e1F1, the skin microbiota after 74 days in e1A1 (74df) shifted markedly toward the environmental water of Fukada Pond (e1F1) the following day. When the fish were moved back to e1A2, the skin microbiota changed markedly the next day, shifting toward the aquaponic environmental water of e1A2. Furthermore, when the fish were returned to e1F2, the skin microbiota shifted toward the e1F2 environmental water microbiota from the day after the transfer.

These results collectively demonstrate that after each environmental transfer, the fish skin microbiota exhibited rapid compositional changes the next day and consistently approached the microbiota plot of the destination environmental water.

Next, we quantified the similarity between the microbial communities on the fish skin pre- and post-transfer using the TDI. The TDI values for pre- and post-transfer were calculated, grouped by translocation interval, and visualized using boxplots (Fig. 3b). The mean TDI values were >0.9 in all groups, indicating high dissimilarity, with values closer to 1 indicating more dissimilar communities and demonstrating that the microbiota composition changed substantially pre- and post-transfer.

In addition, Bray–Curtis PCoA for the entire e1 experiment is shown in Fig. S2. Similar to Fig. 3a, the microbiota of the skin and environmental water in each translocation interval were located close to each other from the first to the last day of each interval. Moreover, the positions of e1F1 and e1F2, which corresponded to transfers to Fukada Pond, were closer to each other than those of e1A1 and e1A2, which corresponded to transfers to the aquaponic system.

Furthermore, the microbiota compositions at the genus, family, class, and phylum levels in e2, as well as Bray–Curtis PCoA, are shown in Fig. S3a and b. The microbiota composition in e2, similar to that in e1, differed among the translocation intervals, and the microbiota composition on the day before transfer and the first day after transfer changed markedly. The PCoA results also resembled those of e1, with skin and environmental water microbiota positioned relatively close to each other within each translocation interval. Thus, in two independent translocation experiments with reproducibility, the fish skin microbiota showed rapid compositional changes by the day after transfer and became more similar to the destination environmental water microbiota than to the pre-transfer state.

### Similarity between fish skin and environmental water microbiota

**Fig 4:**
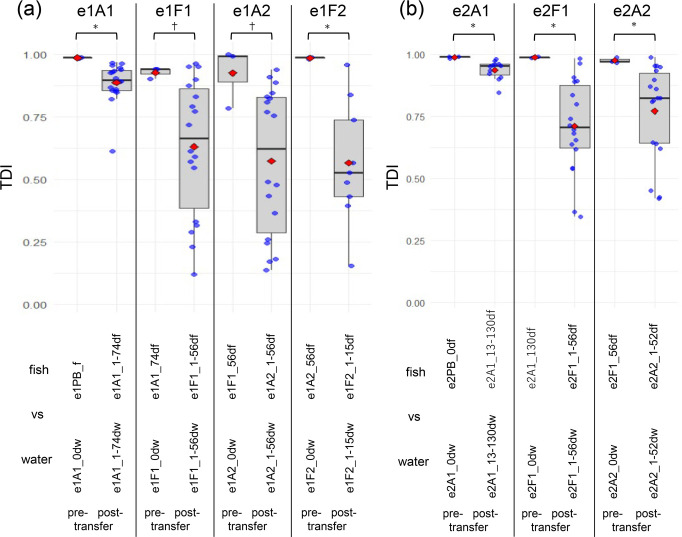
Theta dissimilarity index (TDI; 1 − thetaYC) values comparing microbial community dissimilarity between fish skin and environmental water across translocation events in (**a**) e1 and (**b**) e2. TDI values were calculated by pairing fish skin samples with the corresponding environmental water samples from the destination environment. After transfer, TDI values decreased compared to pre-transfer conditions, indicating that the fish skin microbiota became less dissimilar to the destination water microbiota. Importantly, this reduction demonstrates that post-transfer fish skin microbiota is more similar to the destination water microbiota than to the pre-transfer water microbiota, reflecting a directional shift in community structure rather than complete similarity between the communities. Blue circles represent individual TDI values, red diamonds indicate means, and black lines indicate medians. *P < 0.05, ^†^*P* < 0.1.

The similarity between the microbial communities in environmental water and fish skin was quantified using the TDI. The TDI values pre- and post-translocation were compared for e1 and e2 at each translocation interval (Fig. 4a and b). The calculation of each TDI value was based on the pairing of “fish skin pre-translocation and environmental water at the destination” pre-translocation, and “fish skin and environmental water on each day” post-translocation. In all transfers of e1 and e2, the mean TDI values after transfer decreased compared to those before transfer (e1F1 and e1A2 showed a trend toward significance with *P* = 0.08 and 0.07, respectively, while the others were significant at *P* < 0.05), indicating reduced dissimilarity. Importantly, this reduction in TDI indicates that, after transfer, the fish skin microbiota became more similar to the destination water microbiota than to the pre-transfer water microbiota. The results obtained by grouping the calculated TDI values for each sampling day are presented in Fig. S4a and b. Although daily variations were observed, the mean TDI values were lower than those before the transfer.

Furthermore, comparing aquaponics and Fukada Pond for the first transfer, the TDI values in Fukada Pond were lower, and the group differences were smaller, suggesting that the fish skin microbiota are more likely to resemble the environmental water microbiota in Fukada Pond than in the aquaponics system. In addition, when comparing the first and second transfers for each location, in both e1 and e2, the TDI values were lower, and the group differences were smaller in the second transfer to aquaponics than in the first transfer. In e1, the average of TDI values was also lower in the second transfer to Fukada Pond than in the first. These results suggest that repeated exposure may facilitate increased shifts between fish skin microbiota and environmental water microbiota. However, even though the number of shared taxa between water and skin increased, the microbiota of water and skin never became completely identical after two transfers.

### α-Diversity

**Fig 5:**
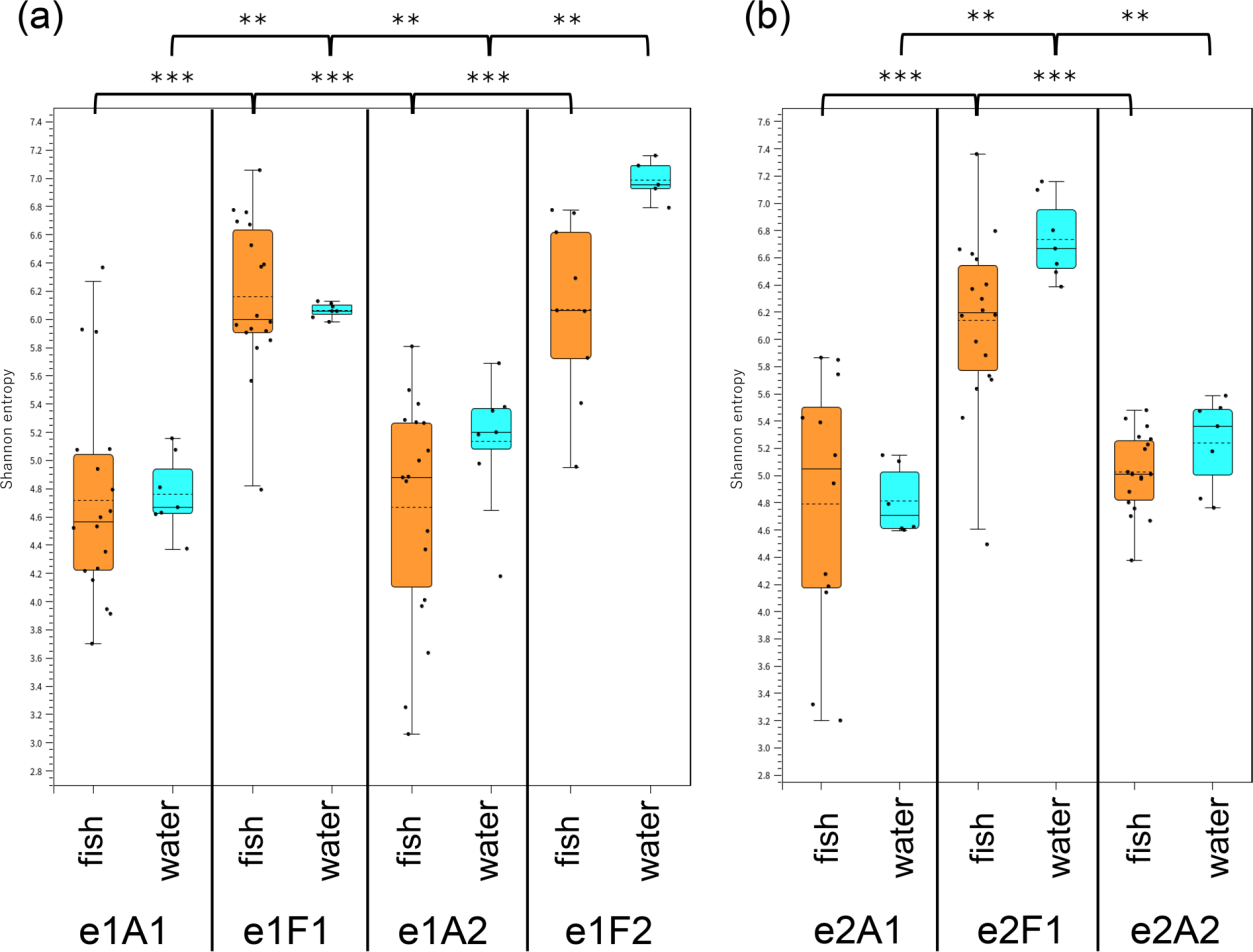
Alpha diversity (Shannon entropy) of water and skin communities across (**a**) e1 and (**b**) e2. Skin alpha diversity is higher in Fukada Pond than in aquaponics and increases upon pond transfer, decreasing upon return to aquaponics. ***P < 0.001, **P < 0.01.

The α-diversity (Shannon entropy) of fish skin and environmental water microbiota at each translocation site in e1 and e2 is shown in Fig. 5a and b. In both experiments, environmental water exhibited significantly higher diversity in Fukada Pond than in the aquaponic system (*P*  <  0.01), and the same trend was observed for fish skin microbiota (*P*  <  0.001). Overall, fish skin diversity increased when moved to Fukada Pond, decreased upon returning to the aquaponic system, and rose again after reintroduction to Fukada Pond, indicating that microbial diversity on the fish surface shifts with habitat changes and reflects the diversity of the surrounding environment.

### Temporal changes in major bacteria

**Fig 6:**
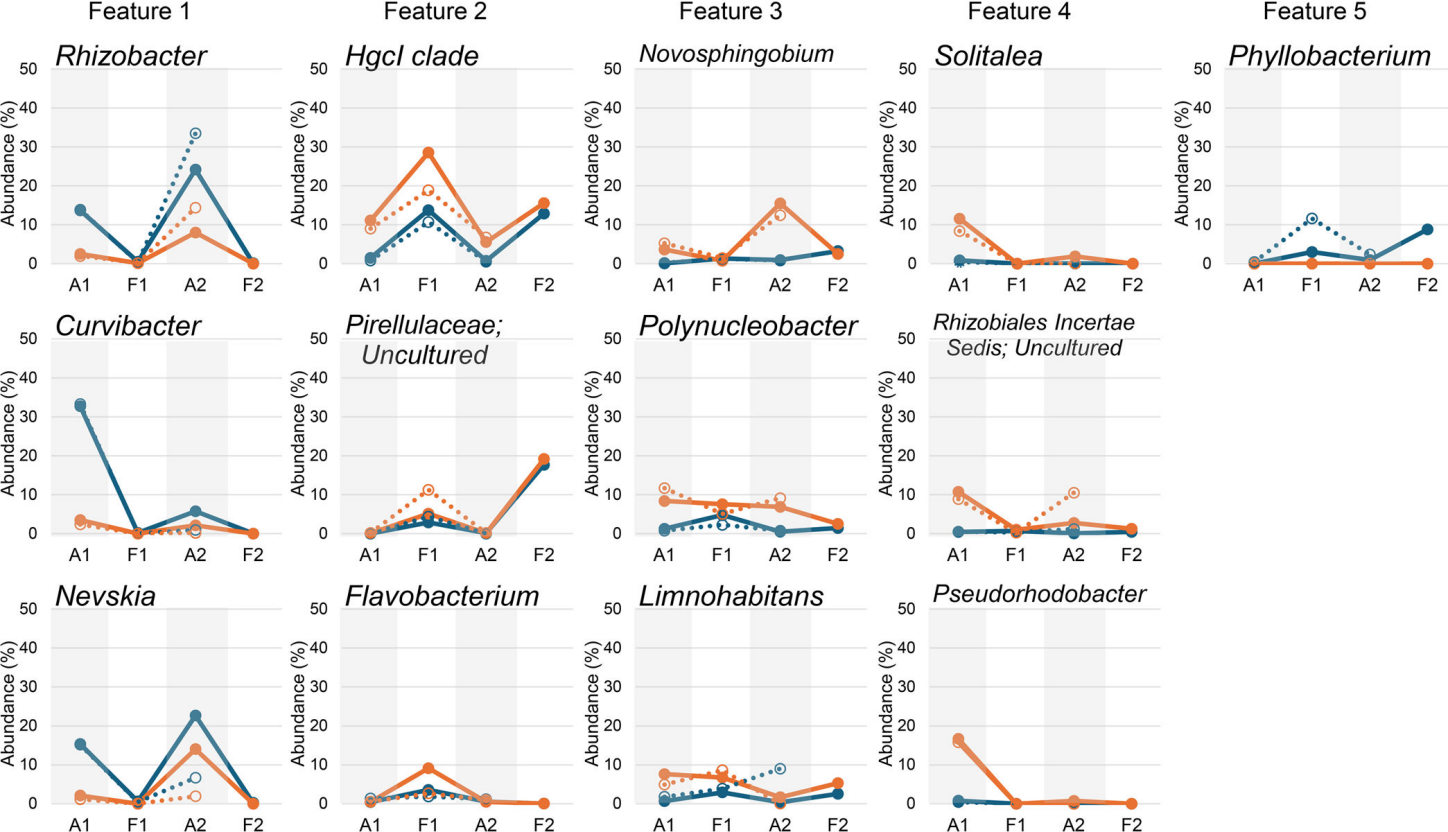
Cross-experiment (e1, e2) comparison of major genera, averaged within each location (A1, F1, A2, F2), showing reproducible behavior patterns. The average abundance (%) of each bacterial species at each transfer location (e1A1, e1F1, e1A2, e1F2 in e1, and e2A1, e2F1, e2A2 in e2) was calculated from Table S4a and b. Representative temporal trajectories of key bacterial genera across moves, illustrating five characteristic behavior patterns. Feature 1, aquaponics-increasing with concordant water-skin behavior; feature 2, pond-increasing with concordant behavior; feature 3, present across all environments; feature 4, water-present but scarce on skin; feature 5, skin-present but scarce in water. The horizontal axis indicates transfer location: A1 (e1A1, e2A1) for the first aquaponics experiment, F1 (e1F1, e2F1) for the first Fukada Pond experiment, A2 (e1A2, e2A2) for the second aquaponics experiment, and Fukada Pond experiment (e1F2 tested, e2F2 not tested). Legend: blue, fish skin; orange, environmental water. Solid lines indicate experiment e1, and dotted lines indicate experiment e2.

Among the microbiota of e1 and e2, temporal changes in the abundance of the top 20 genera (based on the total abundance percentage) for each sampling day are shown in Table S4a and b. Of these 20 genera, 17 were shared between e1 and e2. For the 13 representative genera among these shared taxa, the mean abundances in the fish skin and environmental water for each transfer location in e1 and e2 are shown in Fig. 6. Temporal abundance patterns before averaging for each sampling day are shown in Fig. S5a and b. These bacteria exhibited five characteristic behaviors associated with transfer. Representative examples are as follows.

Feature 1. Taxa increased mainly in the aquaponic system and showed similar temporal patterns between water and skin (e.g., *Rhizobacter*, *Curvibacter*, and *Nevskia*).

Feature 2. Taxa that increased mainly in Fukada Pond and showed similar temporal patterns between the water and skin (e.g., *HgcI clade*, *Pirellulaceae*; *Uncultured*, *Flavobacterium*).

Feature 3. Taxa were detected in both water and skin across all environments (e.g., *Novosphingobium*, *Polynucleobacter*, and *Limnohabitans*).

Feature 4. Taxa were detected mainly in water and rarely on the skin (e.g., *Solitalea*, *Rhizobiales incertae sedis*; *Uncultured*, and *Pseudorhodobacter*).

Feature 5. Taxa that were rare in water but present on skin (e.g., *Phyllobacterium*).

In the taxa categorized as feature 1, the abundance of microbiota on the fish skin and in the water increased during the first transfer to the aquaponic system (A1), decreased when the fish were transferred to Fukada Pond (F1), increased again during the second transfer to the aquaponic system (A2), and decreased again during the second transfer to Fukada Pond (F2). In the taxa categorized as feature 2, in contrast to feature 1, the abundance decreased at A1 and A2 and increased at F1 and F2. Furthermore, the changes in abundance in environmental water and fish skin in features 1 and 2 occurred on nearly the same sampling days, supporting the finding that the microbial communities of fish skin became similar to those of the surrounding water within 1 day after transfer (Fig. S5a and b). These findings are consistent with the results shown in Fig. 3a and b, indicating that the fish skin microbiota changed within 1 day of transfer to different environments.

Because the abundance of the fish skin microbiota increased or decreased synchronously with changes in the environmental water microbiota of the transfer destination, it was inferred that bacteria present in the environmental water of the aquaponic system or Fukada Pond influenced the microbiota composition of the fish skin after transfer, resulting in the replacement of the core microbiota. In contrast, the taxa categorized as feature 4 represent bacteria present in environmental water that do not colonize the fish skin microbiota, indicating that not all environmental bacteria successfully colonize the skin. Although there were taxa whose behaviors differed between environmental water and fish skin, as shown in features 4 and 5, a large proportion of taxa showing similar behavior between environmental water and fish skin (features 1–3) accounted for 80% (16/20) of the top 20 taxa in e1 and 70% (14/20) of the top 20 taxa in e2.

Moreover, a comparison of e1 and e2 showed that all taxa exhibited the same behavior across features 1–5, confirming reproducibility across the two experiments. Overall, these results demonstrated, with reproducibility in two independent experiments, that replacing the rearing environment led to a shift in the major bacteria on fish skin in accordance with the microbiota composition of the destination environmental water. These findings indicate that the microbiota composition of the surrounding water strongly influences many bacterial taxa present on fish skin.

### Major bacterial taxa

Among the bacteria commonly abundant on fish skin in both the aquaponic system and the Fukada Pond, members of the phylum *Proteobacteria* were dominant. In particular, in the aquaponic system, the class *Gammaproteobacteria* accounted for approximately 90% of the skin mucus at all stages (e1A1, e1A2, e2A1, and e2A2) (Fig. 2; Fig. S3a). In Fukada Pond, *Alphaproteobacteria* and *Gammaproteobacteria* were the most abundant within the phylum *Proteobacteria*, followed by *Actinobacteria*, *Bacteroidetes*, and *Planctomycetes*. Bacteria belonging to the *Gammaproteobacteria* that were abundant on fish skin included *Curvibacter*, *Nevskia*, *Rhizobacter*, *Ramlibacter*, *Limnobacter*, *Aeromonas*, and *Polynucleobacter*, which accounted for 14 of the top 20 taxa by abundance.

Comparison of the microbiota between environmental water and fish skin showed that in the aquaponic system, although the environmental water contained approximately 20%–40% *Bacteroidetes* and *Actinobacteria* in addition to *Proteobacteria*, only approximately 10% of these taxa were present on the fish skin. In Fukada Pond, taxa abundant on fish skin tended to be abundant in environmental water.

As shown in Fig. 2 for e1 and Fig. S3a for e2, the genera frequently detected on fish skin after transfer to the aquaponic system included *Curvibacter*, *Nevskia*, *Rhizobacter*, *Ramlibacter*, and *Methylibium* (all within *Gammaproteobacteria*), whereas in Fukada Pond, the abundant genera included the *HgcI clade* (*Actinobacteria*), *Pirellulaceae*; *Uncultured* (*Planctomycetes*), and *Phyllobacterium* (*Alphaproteobacteria*).

**Fig 7:**
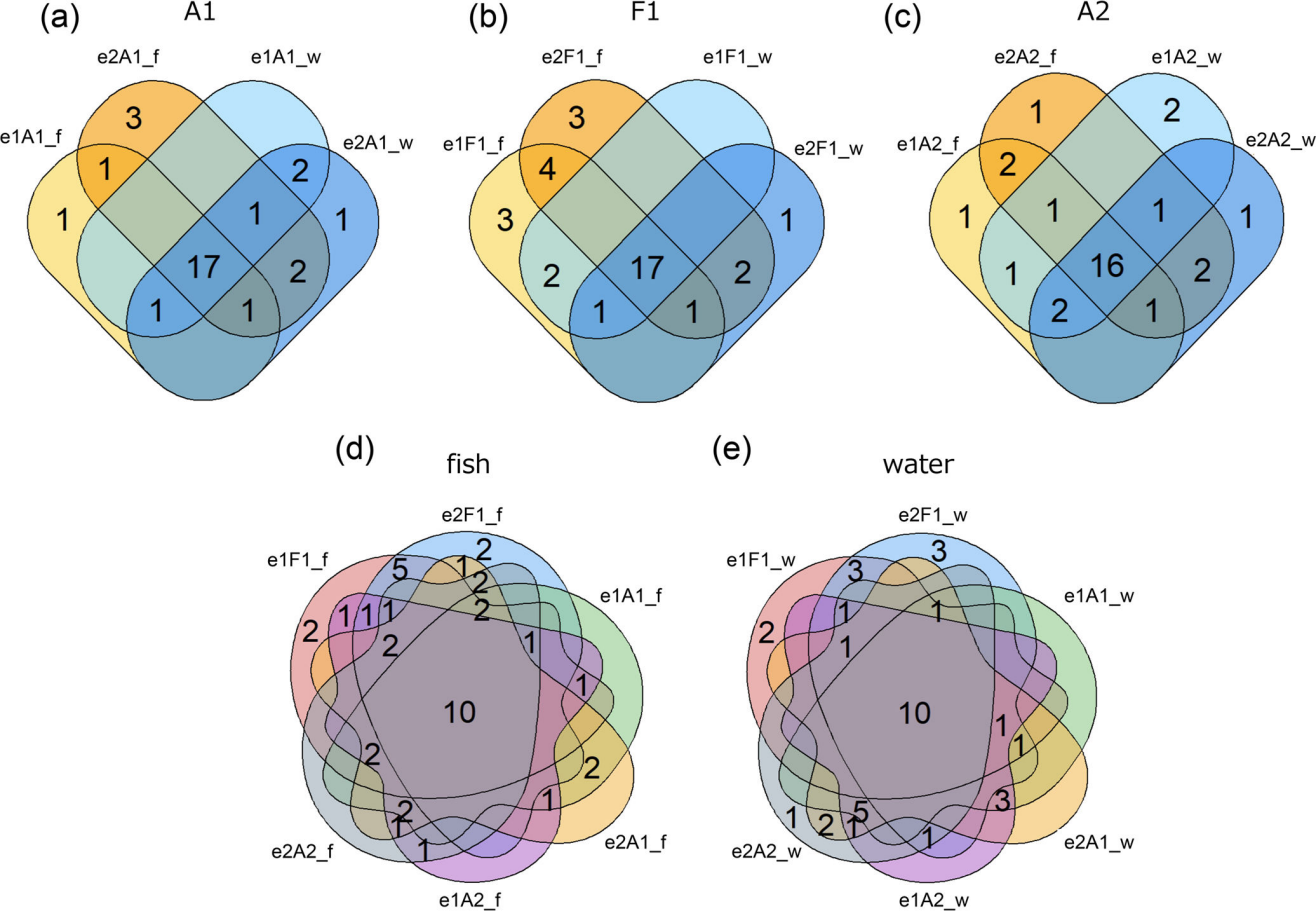
Genera common to both water and skin at each location across e1 and e2 in (**a**) A1, (**b**) F1, and (**c**) A2. Genera are consistently present across all locations throughout the experiments (persistent residents) in (**d**) fish skin and (**e**) water. Relative existence quantities greater than 0.1% are defined as existing. For Venn diagram construction, abundance values <0.1% were binarized to zero. In A1, 56.7% (17/30) of the taxa were shared among all groups, 70.4% (19/27) were shared between the fish skin samples from the two experiments, and 84.0% (21/25) were shared between the environmental water samples. In F1, 50.0% (17/34) were shared across all groups, 66.7% (22/33) were shared among fish skin samples, and 75.0% (18/24) were shared among environmental water samples. In group A2, 51.6% (16/31) were shared across all groups, 71.4% (20/28) shared fish skin, and 70.4% (19/27) shared environmental water. f, fish skin; w, water.

For the top 40 genera (ranked by total abundance percentage) in e1 and e2, Venn diagrams were constructed, showing the bacterial taxa present on the fish skin and in the environmental water for A1 (first aquaponic transfer: e1A1, e2A1), F1 (first Fukada Pond transfer: e1F1, e2F1), and A2 (second aquaponic transfer: e1A2, e2A2) (Fig. 7a through c). Across all translocation intervals, the proportion of taxa shared between fish skin and environmental water in both experiments ranged from 50.0% to 56.7%, demonstrating that many bacterial taxa were similar in both environments across the two independent experiments.

In addition, bacterial taxa that were consistently present on fish skin and in the environmental water at the three transfer locations (A1, F1, and A2) in both e1 and e2 (i.e., bacteria that were consistently present regardless of environmental changes) were extracted (Fig. 7d and e). Ten taxa were consistently present on fish skin, and 10 taxa were consistently present in environmental water. The bacterial taxa that were consistently present on fish skin throughout the experimental period were *Rhizobacter*, *HgcI clade*, *Nevskia*, *Polynucleobacter*, *Limnohabitans*, *Limnobacter*, *Flavobacterium*, *Comamonadaceae*; *Uncultured*, *Delftia*, and *Fluviicola*. The bacterial taxa that were consistently present in the environmental water throughout the experimental period were *Rhizobacter*, *HgcI clade*, *Polynucleobacter*, *Novosphingobium*, *Rhizobiales Incertae Sedis*; *Uncultured*, *Limnobacter*, *Pirellulaceae*; *Uncultured*, *Flavobacterium*, *Mycobacterium*, and *Fluviicola*.

Among these, the taxa that were consistently present on fish skin, but not in the environmental water, were *Nevskia*, *Limnohabitans*, *Comamonadaceae*; *Uncultured*, and *Delftia. Nevskia* was also one of the bacteria detected in high abundance on fish skin after transfer to the aquaponic system, as shown in Fig. 6. Among all examined taxa, the bacteria consistently present in both environmental water and fish skin were *Rhizobacter*, *HgcI clade*, *Polynucleobacter*, *Limnobacter*, *Flavobacterium*, and *Fluviicola*.

## DISCUSSION

Across two independent experiments involving repeated translocations, we demonstrated that the fish skin mucosal microbiota underwent a rapid and pronounced shift within 1 day after transfer and subsequently tended to shift more similarly to the destination water microbiota than to the pre-transfer state. These results collectively demonstrate that fish skin microbiota exhibit marked ecological plasticity and reproducibility in response to surrounding environmental conditions.

### Day-after-transfer shifts in microbiota composition

It is necessary to evaluate how rapidly microbiota changes occur on fish skin, and whether environmental water and skin microbiota become similar. Translocation experiments in fish provide a powerful approach for evaluating time-series changes in microbiota within an exposed (destination) environment. Several studies have reported such translocation experiments, showing that fish skin microbiota are highly plastic and undergo nearly complete microbial community turnover within 6 weeks (17) and that in freshwater-seawater translocation experiments, skin microbiota differ greatly after 1 week (19, 30). However, in all previous studies, fish skin microbiota were examined 1 week or more after transfer, and short-term temporal changes remained unclear.

In the translocation experiments conducted in this study, a comparison of fish skin microbiota immediately before transfer into a different environment and on the day after transfer revealed that substantial changes had already occurred within 1 day, with major taxa being replaced. The immediate changes in the microbiota following translocation indicate that the fish skin microbiota is highly dynamic and largely influenced by the surrounding environmental water microbiota. Demonstrating that environmental water influenced fish skin microbiota within 1 day provides important insights into the interactions between environmental water microbiota and fish skin microbiota.

### Similarity between environmental water and skin microbiota

There are studies both supporting and opposing similarity between environmental water and fish skin microbiota. Several studies comparing environmental water with gut and skin microbiota have shown that fish skin microbiota is more similar to environmental water than to gut microbiota (14, 15, 24, 31). On the other hand, there are also reports that the fish skin microbiota differs from the water microbiota (17–19, 30–34). Northern Pike from Canadian rivers shared less than 30% of ASVs between skin and water (32). Another study showed that Roach transferred between brackish and seawater shared only 2.9% of ASVs (19). Because these studies compared the fish skin microbiome with the microbiome of the water in which the fish lived or to which the fish had been transferred more than several weeks earlier, it remained unclear whether the observed changes, or lack thereof, were due to variability in the fish microbiome itself or to the real effects of environmental and/or host-related factors.

In this study, we conducted time-series sampling from the day after transfer to approximately 2 months thereafter, allowing detailed analysis of changes in both the environmental water and skin microbiota. This made it possible to determine the directionality and variability of skin microbiota changes in terms of whether they became more similar to the water microbiota before or after transfer. Although substantial variability of the similarity between skin and water microbiota was observed, the skin microbiota changed markedly immediately after translocation and became more similar to the post-translocation water than to the pre-translocation water, as suggested by both PCoA plots and TDI comparison (Fig. 4, see also Fig. S2, S3b, and S4). We observed this tendency with the two transfer experiments in which the water microbiota of the aquaponic system was quite different between A1 and A2 because of the 2 months interval between the experiments, supporting the hypothesis that the skin microbiota was influenced by the water microbiota rather than by other factors.

By using the TDI value, a dissimilarity index, as an indicator to evaluate microbiota similarity between fish skin and water, it was possible to quantitatively compare the change with other works. Although it is one of the few available studies, a study comparing the skin mucus, gut contents, and surrounding water microbiota of wild tropical freshwater fish (24), the TDI values were 0.69 for water vs skin and 0.84 for water vs gut, indicating greater similarity between water and skin. The TDI value for water vs fish skin was close to the mean TDI observed in our study during the first transfer to the Fukada Pond (Fig. 4a and b; e1F1: 0.63, e2F1: 0.71).

Although we observed shifts in the fish microbiota and the direction of those shifts after environmental transfer, it remains unclear which environmental factors (e.g., natural vs artificial conditions, temperature, and pH) and/or fish-related factors (e.g., species, age, and number of individuals) drive these patterns (6, 7, 35). Future studies on fish skin and water microbiomes under diverse conditions, using a common index, such as the TDI value, would help clarify these relationships.

### α-Diversity

Previous studies comparing α-diversity of skin and water microbiota have shown that in aquaculture facilities, skin microbiota of Atlantic salmon exhibits lower diversity than tank water and biofilms (15), whereas in wild riverine fish, such as roach, skin microbiota diversity greatly exceeds that of water (19). Comparisons between captive and wild environments indicate that α-diversity of vertebrate skin microbiota tends to decrease under captive conditions (35). In Atlantic salmon, the skin microbiota diversity is higher in natural environments than in hatcheries (17, 36). Our findings align with these results, showing higher skin microbiota diversity in the natural Fukada Pond than in the aquaponic system.

### Influence of environmental water during swabbing

When collecting mucus swabs from fish, the influence of environmental water should be considered; however, it is difficult to completely eliminate this influence. In this study, similarity between the microbiota of environmental water and fish skin differed greatly between the aquaponics system and Fukada Pond, as shown by PCoA, α-diversity, and other results. This suggests that swab samples taken from fish skin mucus do not merely reflect the environmental water adhering to the skin. Moreover, the bacterial taxa classified as features (4 and 5) in Fig. 6 showed inconsistent abundance patterns between environmental water and skin, indicating that the influence of environmental water on skin samples was limited. In previous studies on Atlantic salmon, several genera were shared between the skin mucus and rearing water, suggesting that water-derived strains contribute to skin microbiota formation; however, beta diversity analyses showed clearly distinct clusters between the skin and water microbiota (37, 38).

### Major bacterial taxa

The abundance of bacterial taxa on fish skin differed between the aquaponic system and Fukada Pond. In the aquaponic system, the phylum *Proteobacteria* (primarily the class *Gammaproteobacteria*) accounted for nearly 90% of the community, which is consistent with reports that *Gammaproteobacteria* are abundant in freshwater fishes (6, 9, 14, 15, 39). In Fukada Pond, in addition to *Proteobacteria* (classes *Alphaproteobacteria* and *Gammaproteobacteria*), the phyla *Actinobacteriota*, *Bacteroidota*, and *Planctomycetota* were abundant. Previous studies have reported that in teleost skin, bacteria belonging to *Proteobacteria* and *Bacteroidota* are dominant (40, 41), and in the skin microbiota of Atlantic salmon, *Proteobacteria* are overwhelmingly predominant, followed by small proportions of *Firmicutes*, *Actinobacteriota*, and *Bacteroidota* (17). These findings suggest that although the major bacterial composition in our study resembles that reported previously, the dominant taxa differ depending on habitat conditions.

In this study, the bacterial taxa consistently present in both the environmental water and fish skin included *Rhizobacter*, *HgcI clade*, *Polynucleobacter*, *Limnobacter*, *Flavobacterium*, and *Fluviicola*. Among these, *HgcI clade* is a freshwater planktonic bacterium (42), and *Polynucleobacter* is a lineage of planktonic freshwater bacteria (43). *Fluviicola* has been reported as part of the core gut microbiota of zebrafish (*Danio rerio*) (44), and *Flavobacterium* has been reported as an intestinal bacterium in medaka (*Oryzias latipes*) (45). The findings suggest that these taxa reside in both fish skin and environmental water, potentially forming a shared microbial network that links the host and its environment.

### Future directions

In this transplantation experiment, the skin core microbiota likely rapidly reconstructed under the influence of the destination water microbiota. Demonstrating such short-term plasticity represents a crucial step toward predicting and improving fish health. Future research faces two major challenges: elucidating interactions among environmental water microbiota, skin microbiota, and the fish host, and clarifying the relationship between fish’s health and skin microbiota.

First, while metabolic analysis of environmental samples presents technical challenges, integrating host mucus transcriptome and microbial metabolic profiling could contribute to elucidating the host-microbe interactions underlying microbial community restructuring.

The second challenge is defining a “healthy” skin microbiota. In aquaculture systems, tank biofilms have been shown to resemble fish skin microbiomes and function as microbial reservoirs (15, 16). These findings suggest that microbial management in artificial environments like aquaculture could contribute to maintaining fish health and improving rearing conditions. Approaching rearing conditions closer to natural waters or fish skin microbiomes may lead to better outcomes.

Future multi-omics analyses will be crucial for elucidating these relationships and guiding microbiome-based management strategies.

## Data Availability

The data have been deposited with links to BioProject accession number PRJDB39956 in the DDBJ BioProject database. Read data were deposited under accession numbers DRR898747–DRR898902.

## SUPPLEMENTAL MATERIAL

Supplemental figures and tables spectrum.00522-26-s0001.docx Fig. S1 to S5, Table S1, and Table S4.

Fig. S2a spectrum.00522-26-s0002.xlsx The OTU table of e1.

Fig. 2b spectrum.00522-26-s0003.xlsx The OTU table of e2.

Fig. S3 spectrum.00522-26-s0004.xlsx Read counts for each sample in e1 and e2.

ASM does not own the copyrights to Supplemental Material that may be linked to, or accessed through, an article. The authors have granted ASM a non-exclusive, world-wide license to publish the Supplemental Material files. Please contact the corresponding author directly for reuse.
