# Supplementary material for: Rapid and reversible fish skin mucosal microbiota shift toward environmental microbial communities within a day
Source: Microbiol Spectr. 2026 Jul 16;14(8):e00522-26. doi: 10.1128/spectrum.00522-26 (PMC13435700; doi:10.1128/spectrum.00522-26)
Supplement: Supplementary file 1 [file spectrum.00522-26-s0001.docx]

**Supplementary Figure S1.** Overview of the aquaponics system and Fukada Pond at the study site.

**Supplementary Figure S2.** Principal coordinate analysis (PCoA, Bray–Curtis) for e1, showing large day-after shifts after each move. PB, aquaponics, and Fukada Pond are separated and skin and water in each translocation were closely related.

Each plot indicate: e1PB water; gray, e1PB fish; white, e1A1 water; blue, fish; light blue, e1F1 water; pink, fish; light pink, e1A2 water; yellowish brown, fish; light yellowish brown, e1F2 water; green, fish; light green.

**Supplementary Figure S3.**

(a) Community composition (genus/family/class/phylum) of water and fish skin across all stages of e2 (e2PB → e2A1 → e2F1 → e2A2).

(b) PCoA (Bray–Curtis) for e2, showing the same qualitative dynamics as e1.

Each plot indicate: e2PB water; gray, e2PB fish; white, e2A1 water; blue, e2A1 fish; light blue, e2F1 water; pink, e2F1 fish; light pink, e2A2 water; yellowish brown, e2A2 fish; light yellowish brown.

**Supplementary Figure S4.** TDI values grouped by sampling day after each transfer in (a) e1 and (b) e2. Calculated TDI values are indicated by blue circles. Mean (red diamond) and median (black line) decrease after transfer.

**Supplementary Figure S5.** Temporal trajectories of key bacterial genera by sampling date across translocation in (a) e1 and (b) e2, illustrating five characteristic behavior patterns.

**Supplementary Table S1.** Sampling schedule and metadata for e1 and e2.

**Supplementary Table S4.** Temporal abundances of the top 20 taxa across (a) e1 and (b) e2 sampling days.

(a)

(b)
